# Supplementary figures and images for: Individual differences of limitation to extract beat from Kuramoto coupled oscillators: Transition from beat-based tapping to frequent tapping with weaker coupling
Source: PLoS One. 2023 Oct 9;18(10):e0292059. doi: 10.1371/journal.pone.0292059 (PMC10561847; doi:10.1371/journal.pone.0292059)

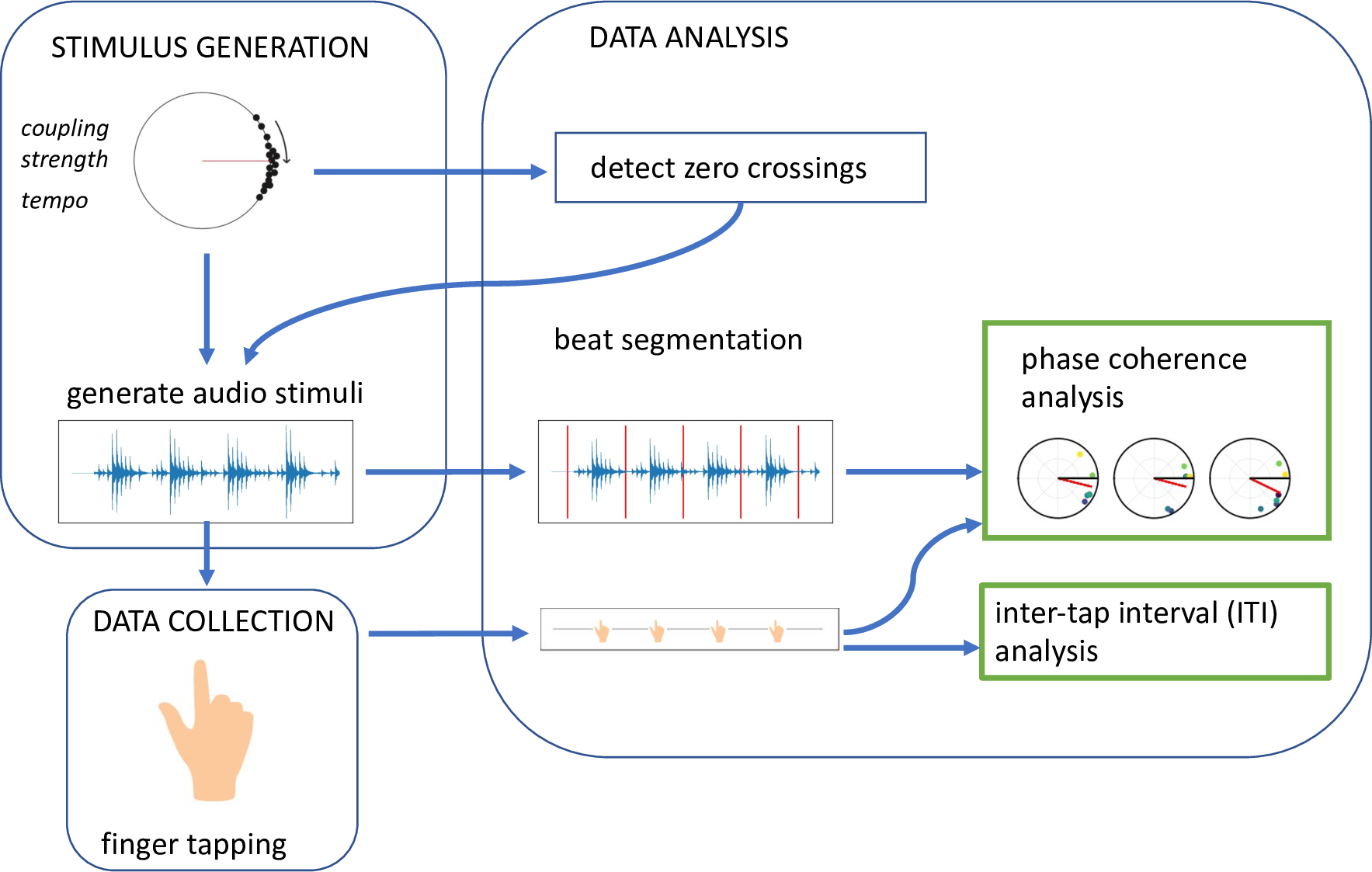

Supplement: S1 Fig — Left top panel (‘STIMULUS GENERATION’): Kuramoto model was used to generate audio stimuli with 40 coupled oscillators with different coupling strength conditions (strong, medium, weak for Exp.1 and strong, medium, weak, none for Exp. 2) and various base tempo (90–110 BPM for Exp.1 and 72–119 BPM for Exp.2). Left bottom panel (‘DATA COLLECTION’): The generated audio stimuli were used for tapping data collection. Right panel (‘DATA ANALYSIS’): Kuramoto model behaviors were used to determine beat windows which were compared against tap timing, resulting in the phase coherence regarding tapping onsets, stimulus onsets, and tap and stimuli relationships. Inter-tap intervals were also analyzed to see its frequency compared to the beat bin size, and adaptive patterns over stimulus sequence duration in a given trial. For more information see S1 File. (TIF) [file pone.0292059.s001.tif]
