# Supplementary material for: Individual differences of limitation to extract beat from Kuramoto coupled oscillators: Transition from beat-based tapping to frequent tapping with weaker coupling
Source: PLoS One. 2023 Oct 9;18(10):e0292059. doi: 10.1371/journal.pone.0292059 (PMC10561847; doi:10.1371/journal.pone.0292059)
Supplement: S2 Fig — (Left) Histogram of tap dispersion. The red vertical line indicates the criterion to separate ‘dense’ and ‘sparse’ tap responses. (Right, top row) Stimulus wave form (blue) with beat windows (orange), beat centers (green), and a sample individual tap responses (red). (Right bottom row). Examples of “phase portraits” for the intervals of each subject’s tap response (red) and stimulus beat centers (blue) for a tap response part of the cluster 1 as regular beat tapping (A.) and two frequent tap responses part of the cluster 3 that was classified as ‘dense’ (B.) and ‘sparse’ (C.) respectively. (PDF) [file pone.0292059.s002.pdf]

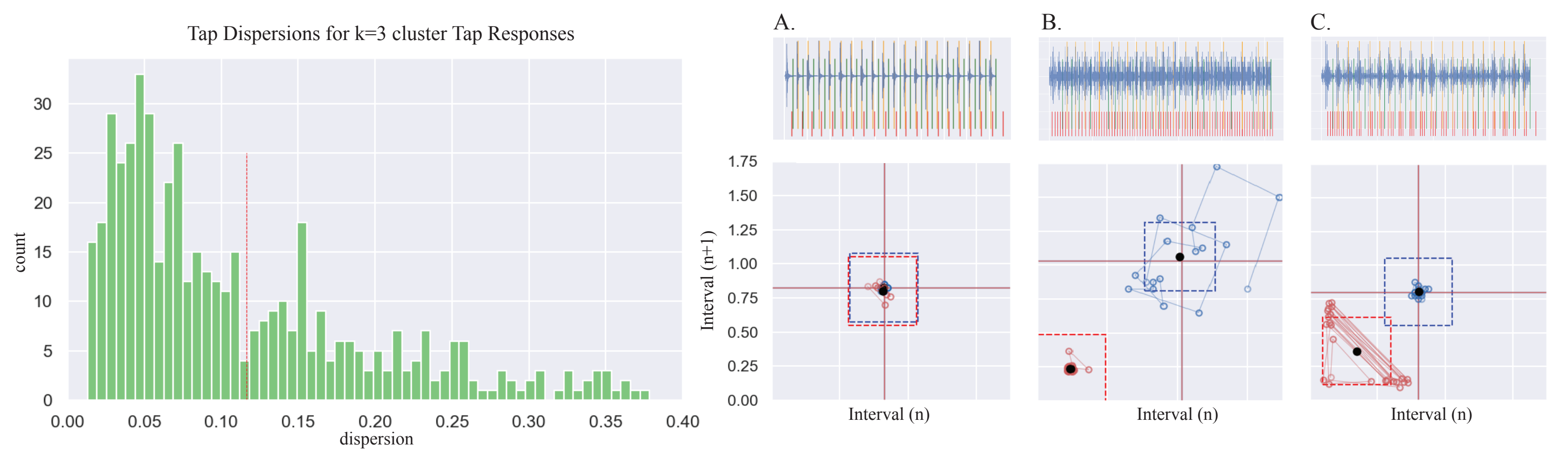
Tap Dispersions for k=3 cluster Tap Responses
count
dispersion
0
5
10
15
20
25
30
0.00
0.05
0.10
0.15
0.20
0.25
0.30
0.35
0.40
A.
B.
C.
Interval (n+1)
Interval (n)
Interval (n)
Interval (n)
0.00
0.25
0.50
0.75
1.00
1.25
1.50
1.75
