# Supplementary material for: Individual differences of limitation to extract beat from Kuramoto coupled oscillators: Transition from beat-based tapping to frequent tapping with weaker coupling
Source: PLoS One. 2023 Oct 9;18(10):e0292059. doi: 10.1371/journal.pone.0292059 (PMC10561847; doi:10.1371/journal.pone.0292059)
Supplement: S1 Table — Note. Significance levels are indicated as *** p < .001, ** p < .01, * p < .05, ns = not significant. For post-hoc results: S = strong, M = medium, W = weak, N = none, T1-7 = tapping section 1–7, >>> = significantly greater (p < .001), >> = significantly greater (p < .01), > = significantly greater (p < .05), ∼ = not significantly different. (DOCX) [file pone.0292059.s003.docx]

|  |  | Mean nITI | | | | | SD of nITI | | | | |
| --- | --- | --- | --- | --- | --- | --- | --- | --- | --- | --- | --- |
| Group | Factor | (dfn, dfd) | F | p | η_G_2 | Significance and Post-hoc test results | (dfn, dfd) | F | p | η_G_2 | Significance and Post-hoc test results |
| Regular | Coupling | F(3, 48) | 2.86 | 0.047 | 0.035 | * | F(3.48) | 27.65 | < 0.001 | 0.22 | *** |
|  |  |  |  |  |  | S > M, M > N, W >> N |  |  |  |  | (S, M, W) <<< N |
|  |  |  |  |  |  |  |  |  |  |  | S <<< W, S << M |
|  |  |  |  |  |  |  |  |  |  |  | M < W |
|  | Section | F(6, 96) | 6.79 | < 0.001 | 0.13 | *** | F(6, 96) | 15.68 | < 0.001 | 0.24 | *** |
|  |  |  |  |  |  | T1 >>> (T4, T5, T6, T7) |  |  |  |  | T1 >>> (T3, T4, T5, T6, T7) |
|  |  |  |  |  |  | T2 >>> (T5, T6, T7) |  |  |  |  | T2 >> (T3, T4, T5, T6, T7) |
|  |  |  |  |  |  | T3 >> (T6, T7) |  |  |  |  | T3 >> (T6, T7) |
|  |  |  |  |  |  | T4 > T6 |  |  |  |  | T4 > T7 |
|  | Coupling x Section | F(18, 288) | 3.91 | < 0.001 | 0.1 | *** | F(18, 288) | 2.26 | 0.0028 | 0.068 | ** |
|  |  |  |  |  |  | N: T4 > T6, T5 > T7 |  |  |  |  | M: T1 > T3, T1 >>> T4, T1 > T5 |
|  |  |  |  |  |  |  |  |  |  |  | W: T1 > T7, T2 > T4 |
| Hybrid | Coupling | F(3, 69) | 165.26 | < 0.001 | 0.73 | *** | F(3.69) | 18.86 | < 0.001 | 0.16 | *** |
|  |  |  |  |  |  | (S, M, W) >>> N, S > M |  |  |  |  | (S, M, W) <<< N |
|  |  |  |  |  |  |  |  |  |  |  | S <<< (M, W) |
|  | Section |  |  |  |  | Ns | F(6,138) | 10.25 | < 0.001 | 0.11 | *** |
|  |  |  |  |  |  |  |  |  |  |  | T1 >>> (T2, T3, T4, T5, T6, T7) |
|  |  |  |  |  |  |  |  |  |  |  | T1 > (T5, T6) |
|  | Coupling x Section | F(18, 414) | 7.01 | < 0.001 | 0.11 | *** |  |  |  |  | Ns |
|  |  |  |  |  |  | S: T5 >>> T4 >>> T6 >>> T2 >>> T3 >>> T1 |  |  |  |  |  |
|  |  |  |  |  |  | N: T2 >> T7, T3 > T7 |  |  |  |  |  |
| Fast | Coupling | F(3, 24) | 126.16 | < 0.001 | 0.92 | *** | F(3,24) | 6.8 | 0.0018 | 0.26 | ** |
|  |  |  |  |  |  | S >>> (M, W) |  |  |  |  | (M, W) >>> S |
|  |  |  |  |  |  | (S, M, W) >>> N |  |  |  |  | (M, W) >>> N |
|  | Section |  |  |  |  | Ns |  |  |  |  | Ns |
|  | Coupling x Section | F(18, 144) | 2.8 | < 0.001 | 0.061 | *** | F(18,144) | 2.18 | 0.006 | 0.11 | *** |
|  |  |  |  |  |  | N: T1 > T6 |  |  |  |  | S: T1 > (T3, T5) |
