# Supplementary material for: Individual differences of limitation to extract beat from Kuramoto coupled oscillators: Transition from beat-based tapping to frequent tapping with weaker coupling
Source: PLoS One. 2023 Oct 9;18(10):e0292059. doi: 10.1371/journal.pone.0292059 (PMC10561847; doi:10.1371/journal.pone.0292059)
Supplement: S2 Table — (DOCX) [file pone.0292059.s004.docx]

|  |  |  |  | Dense |  | Sparse |
| --- | --- | --- | --- | --- | --- | --- |
|  | **Coupling** | **Proportion of Cluster 3 in Total tap trials (%)** | **Proportion of dense tap in Total tap trials (%)** | **Mean (SD)**  **raw ITI (s)** | **Proportion of sparse tap in Total tap trials (%)** | **Mean (SD)**  **raw ITI (s)** |
| **Regular** | Strong | 0 | NA | NA | NA | NA |
|  | Medium | 0 | NA | NA | NA | NA |
|  | Weak | 1.9 | NA | NA | 1.9 (39.0) | 0.45 (0.079) |
|  | None | 29.4 | 15.0 (15.4) | ﻿0.589 (0.16) | 14.4 (13.2) | ﻿0.598 (0.19) |
| **Hybrid** | Strong | 0 | NA | NA | NA | NA |
|  | Medium | 6.3 | 0.42 (1.9) | 0.39 (0.0) | 5.8 (11.3) | 0.41 (0.066) |
|  | Weak | 5.0 | 1.25 (4.2) | 0.36 (0.10) | 3.8 (8.2) | 0.39 (0.11) |
|  | None | 78.3 | 37.5 (18.7) | 0.46 (0.15) | 40.8 (20.8) | 0.50 (0.18) |
| **Fast** | Strong | 0 | NA | NA | NA | NA |
|  | Medium | 85.6 | 31.3 (19.0) | 0.23 (0.015) | 52.5 (22.8) | 0.31 (0.033) |
|  | Weak | 94.4 | 37.5 (22.8) | 0.22 (0.011) | 56.3 (26.4) | 0.29 (0.037) |
|  | None | 98.9 | 70.0 (33.2) | 0.23 (0.020) | 28.8 (31.4) | 0.29 (0.061) |
