# Supplementary material for: Individual differences of limitation to extract beat from Kuramoto coupled oscillators: Transition from beat-based tapping to frequent tapping with weaker coupling
Source: PLoS One. 2023 Oct 9;18(10):e0292059. doi: 10.1371/journal.pone.0292059 (PMC10561847; doi:10.1371/journal.pone.0292059)
Supplement: S3 Table — Note. Significance levels (S) are indicated as *** p < .001, ** p < .01, * p < .05. (DOCX) [file pone.0292059.s005.docx]

|  | **Dense Taps** | | | | | **Sparse Taps** | | | | |
| --- | --- | --- | --- | --- | --- | --- | --- | --- | --- | --- |
|  | Estimate | Standard Error | t-statistic | P value |  | Estimate | Standard Error | t-statistic | P value |  |
| **ITI** |  |  |  |  |  |  |  |  |  |  |
| predictors |  |  |  |  |  |  |  |  |  |  |
| (Intercept) | 0.203 | 0.083 | 2.452 | 0.015 | * | 0.352 | 0.115 | 3.073 | 0.002 | ** |
| R | 0.183 | 0.337 | 0.545 | 0.586 |  | 0.013 | 0.355 | 0.037 | 0.971 |  |
| Tempo | 0.000 | 0.001 | 0.025 | 0.980 |  | 0.000 | 0.001 | -0.085 | 0.932 |  |
| Fast grp | 0.496 | 0.182 | 2.728 | 0.007 | ** | 0.641 | 0.150 | 4.260 | 0.000 | *** |
| Hybrid grp | 1.121 | 0.432 | 2.596 | 0.010 | ** | 0.650 | 0.202 | 3.224 | 0.001 | ** |
| R x Tempo | -0.001 | 0.004 | -0.369 | 0.712 |  | -0.001 | 0.004 | -0.267 | 0.790 |  |
| R x Fast grp | -0.265 | 2.093 | -0.127 | 0.899 |  | -1.174 | 0.655 | -1.793 | 0.075 |  |
| R x Hybrid grp | -3.955 | 6.399 | -0.618 | 0.537 |  | 0.738 | 1.389 | 0.531 | 0.596 |  |
| Tempo x Fast grp | -0.003 | 0.002 | -1.295 | 0.196 |  | -0.007 | 0.002 | -3.275 | 0.001 | ** |
| Tempo x Hybrid grp | -0.010 | 0.006 | -1.669 | 0.096 |  | -0.006 | 0.003 | -2.303 | 0.022 | * |
| R x Tempo x Fast grp | 0.000 | 0.027 | -0.009 | 0.993 |  | 0.014 | 0.008 | 1.760 | 0.080 |  |
| R x Tempo x Hybrid grp | 0.042 | 0.092 | 0.461 | 0.646 |  | -0.008 | 0.017 | -0.486 | 0.627 |  |
|  |  |  |  |  |  |  |  |  |  |  |
| **Dispersion** |  |  |  |  |  |  |  |  |  |  |
| predictors |  |  |  |  |  |  |  |  |  |  |
| (Intercept) | 0.045 | 0.016 | 2.816 | 0.005 | ** | 0.202 | 0.124 | 1.629 | 0.105 |  |
| R | 0.041 | 0.066 | 0.629 | 0.530 |  | 0.287 | 0.385 | 0.746 | 0.457 |  |
| Tempo | 0.000 | 0.000 | -0.485 | 0.628 |  | 0.001 | 0.002 | 0.457 | 0.648 |  |
| Fast grp | 0.083 | 0.035 | 2.336 | 0.020 | * | -0.106 | 0.163 | -0.649 | 0.517 |  |
| Hybrid grp | 0.109 | 0.084 | 1.296 | 0.196 |  | 0.061 | 0.218 | 0.278 | 0.781 |  |
| R x Tempo | 0.000 | 0.001 | 0.299 | 0.765 |  | -0.005 | 0.005 | -1.066 | 0.288 |  |
| R x Fast grp | -0.519 | 0.407 | -1.274 | 0.204 |  | 0.906 | 0.710 | 1.276 | 0.204 |  |
| R x Hybrid grp | -0.692 | 1.245 | -0.556 | 0.579 |  | 0.919 | 1.506 | 0.610 | 0.542 |  |
| Tempo x Fast grp | -0.001 | 0.000 | -1.693 | 0.092 |  | 0.000 | 0.002 | 0.130 | 0.896 |  |
| Tempo x Hybrid grp | -0.001 | 0.001 | -0.989 | 0.323 |  | -0.001 | 0.003 | -0.376 | 0.707 |  |
| R x Tempo x Fast grp | 0.005 | 0.005 | 0.942 | 0.347 |  | -0.006 | 0.009 | -0.722 | 0.471 |  |
| Tempo x Hybrid grp | 0.006 | 0.018 | 0.348 | 0.728 |  | -0.007 | 0.018 | -0.408 | 0.684 |  |
