# Supplementary material for: Individual differences of limitation to extract beat from Kuramoto coupled oscillators: Transition from beat-based tapping to frequent tapping with weaker coupling
Source: PLoS One. 2023 Oct 9;18(10):e0292059. doi: 10.1371/journal.pone.0292059 (PMC10561847; doi:10.1371/journal.pone.0292059)
Supplement: S1 File — (DOCX) [file pone.0292059.s006.docx]

# Supporting Information

## Experimental Flow Chart

S1 Fig summarizes the flow chart of this study, common across both experiments, showing the relationship between the coupled-oscillator generative model with 40 oscillators for sound stimulus production, and tapping data collection and analyses. The coupling was parameterized with different strengths and intrinsic frequencies centered around a designated tempo range (90-110 BPM for Experiment 1 and 72-119 BPM for Experiment 2). As shown in S1 Fig, the average angle, ϕ , from the generative model at each time step was used to determine beat windows using the beat window segmentation algorithm extracted the referent beat centers. The tapping data were analyzed in terms of ITI for each tempo and coupling condition. Thereafter, their tap timing data were circularly mapped onto the beat windows in order to perform phase coherence analysis (per beat region) which were compared to the aforementioned stimuli’s phase coherence.

## Beat Window Segmentation of the Auditory Stimuli

We used a series of summary statistics and time-domain signal processing techniques to extract “beat windows” from the audio stimuli in order to be able to compare participant and stimulus onsets with respect to a “beat center”. This segmentation of participant taps and stimulus onsets is a necessary step in order to be able to derive the complex order parameters needed for phase coherence analysis. The algorithm for extracting these beat windows from the auditory stimuli is as follows:

1) We performed amplitude peak picking of the local maxima of the average phase coherence angle ϕ_model_(t) as generated at each sampling interval during the synthesis of the stimuli. This was performed by comparing neighboring samples based on specified conditions for their height, prominence, width, threshold, and distance from one another (these parameters were selected heuristically). This technique of peak picking is suitable to capture the peaks not only for the strongly coupled waveforms containing a visible periodic amplitude envelope but also for the weakly coupled waveforms in which a well-defined periodic amplitude envelope is less salient.

2) These beat windows were shifted so that the center of the beat was situated in the center of the window. We use the convention that the center of the beat window is at 0 degrees with the convention that positive radians are in the counterclockwise direction and negative radians in the clockwise direction.

Previous research in P-center determination, has shown that the P-center is typically found at the amplitude peak for short sounds with fast attack times. This beat window segmentation algorithm positions the center of the beat at the center of the waveform peak which is also associated with the aggregate density of sound onsets in time.

## Characterization of Frequent tapping: delineating between “dense” and “sparse” tapping patterns

Phase portraits, depicting a two-dimensional depiction of sequential tap ITI or stimulus IOI, were generated for each of the tap responses and stimulus onsets. The tapping responses in the cluster 3 (frequent tapping) were further separated by a criterion of their dispersion into ‘dense’ and ‘sparse’ responses as illustrated in the histogram in S2 Fig. Smaller dispersion indicates reduced tap interval variability, while larger dispersion indicate increased variability. Lastly, for each tap response within the cluster 3, we calculated a 2D centroid point made out of the phase portrait for both the taps and the stimulus beat centers. Then, we calculated the centroid difference which is simply the length between the tap centroid and the stimulus centroid. Centroid difference in the frequent tapping trials was used to verify whether the close distance between stimulus beat center and tap center is due to the regular beat tapping pattern like in example in S2 Fig A. Dispersion data were further analyzed further in terms of how dense and sparse tapping trials differed (see S2 Table).

*Phase Portraits*

ITI phase portraits were generated for the second half of each subject’s tap response for each stimuli sequence to capture tapping patterns without the initial adaptation-related variations. Phase portraits show the ITI of each subject’s tap in relation to their previous tap as plotted on a plane along two dimensions; included in these plots is the placement of each stimulus beat center that is treated accordingly. The mean ITI and mean beat center is represented as a black dot. RMS dispersion (variance around mean) was calculated for each distribution. S2 Fig A shows an example of these phase portraits for a ‘regular’ tapping response, while S2 Fig B and S2 Fig C show two frequent tapping responses. IOI(n) indicates the current ITI or IOI of the subject’s tap response (red) of the stimulus’ beat center and the IOI(n+1) indicates the next ITI or IOI. Plot A shows a ‘regular’ tap response: tap dispersion is low and the centroid of the tap responses and stimulus beat centers overlap. Plot B shows an example of a ‘fast’ tapping response where tap dispersion is low but the centroid of the tap distribution is much lower than that of the stimulus beat centers which has large beat center dispersion. Plot C shows another example of a ‘fast’ tapping response; here, the centroid of the tap distribution is much lower than the stimulus beat centers but dispersion of the taps is high and regular which suggests they were tapping with more rhythmic variation.

*Proportion of Dense and Sparse Tapping Responses*

S2 Table shows the percentage of trials and standard deviation that were classified into the dense or sparse tap responses within the cluster 3 (as a percentage of the total) for each coupling condition and participant group. The groups exhibited substantially different proportion of trials that were classified into the ‘dense’ and ‘sparse’ tap responses. Also the table indicate the average raw ITI, which generally shows shorter values for Fast group, followed by Hybrid, and Regular group. Group differences assessed by t-tests are described in the main text.

## Effect of Tempo and Group on the raw ITI and Dispersion: Multiple Regression

Linear regression was used to determine if raw ITI and mean tap dispersion for both the dense and sparse tap responses could be predicted by phase coherence, stimulus tempo, and participant category (mean tap dispersion ~ R*tempo*participant-category). The results of raw ITI are described in the main text, while details are indicated in the S3 Table.

The same model was applied for dispersions. For the dense tap dispersions, the regression was statistically significant (*R^2^* = 0.1666, *F*(11, 283) = 5.144, *p* << 0.0001). The dense tap responses in the Fast group (*B* = 0.083, *p* = 0.021) significantly predicted mean tap ITI for the dense trials. The sparse tap responses did not show any significant main effects or interactions. Within the sparse tap dispersions, the regression was also statistically significant (*R^2^* = 0.1026, *F*(11, 192) = 1.995, *p* = 0.0308), but with no significant main effects or interactions. For the dispersion regression analysis, only the dense tap responses in the Fast group significantly predicted mean tap dispersion, likely reflecting the substantially peculiar tapping approaches in this group.
