# Supplementary material for: Individual differences of limitation to extract beat from Kuramoto coupled oscillators: Transition from beat-based tapping to frequent tapping with weaker coupling
Source: PLoS One. 2023 Oct 9;18(10):e0292059. doi: 10.1371/journal.pone.0292059 (PMC10561847; doi:10.1371/journal.pone.0292059)
Supplement: Attachment — Submitted filename: reviewer-comments.docx [file pone.0292059.s008.docx]

Dear reviewers,

We appreciate your detailed and insightful comments. We think that our manuscript has improved by taking into account your criticisms and suggestions. Below we respond to each of your comments and note where and how those responses are handled in the revised manuscript. Revisions to the manuscript and our responses to comments below are in green font.

Reviewer #1: The manuscript reads much better and all my comments and concerns have been addressed. The study design, results and implications are much more clearly communicated. The work will be an excellent contribution to the research field.

Below is a list of suggestions for minor revisions/further improvements:

- The abstract needs to be rewritten to improve clarity and better reflect the revisions that have been made to the main manuscript.

Thank you for this comment. We have revised the abstract to better accommodate the revisions in this manuscript.

p. 3 “sound sequences that varied the degree of such deviations systematically”. I would consider using “spread in multiple sound onsets” instead of deviations here (it is not clear what the deviations deviate from).

Thank you for this suggestion. We have changed the wording as suggested.

p. 3 I suggest changing the following sentence in the second paragraph to: “For example, listeners’ tolerance for pulse attribution is nearly twice as large as their threshold for detecting simple irregularity…”

We have changed the wording as suggested

p. 5, line 9: Change “not only the” to “is not the only”

We have changed the wording as suggested

p. 5, last paragraph, first line: Change “which” to “with”. This sentence is generally a bit difficult to parse.

We have changed the wording as suggested

p. 6: The third hypothesis could be more clearly formulated. The mention of auditory features is particularly confusing (auditory features are not discussed anywhere else in the manuscript).

Thank you for pointing this out. We removed any reference to auditory features and we added “When confronted with multiple inter-onset intervals” to better specify the stimulus characteristic we are alluding to.

p. 13, line 2: Perhaps say participants instead of group since the “group” consists of only 2 people ϑ

Thanks for this comment, we have changed the wording to ‘participants’.

p. 17-19. Parts of the text under “Data analysis” on p. 17 is repeated on pages 18 and 19.

Thank you for pointing out the potential redundancy. However, we could not clearly identify the parts in question. We made a few wording changes in p. 18-19 for clarity. We would appreciate if further changes are necessary.

p. 18, lines 3-4: Consider replacing “frequent tapping responses” with “high frequency tapping responses” (the former could be misunderstood)

We changed the wording as suggested.

p. 19, second paragraph: Change the following sentence to: “In the Fast tapping group, the majority of tap responses for the three weaker coupling conditions belongs to the frequent tapping cluster, increasing from 85% for the medium, to 94% for the weak, to 99%... “

We changed the wording as suggested.

p. 21 second paragraph – make clear that the shorter ITIs is mainly driven by a switch to frequent tapping? (it’s probably not a gradual thing)

Thank you for noticing an interesting potential connection here. The observation that the within-segment mean nITI in the Regular group is approaching to 0.75 on average for later tapping sections (Figure 4B, Regular group) in the none-coupling condition indeed coincided with the observation that the longest nITI in the cluster 3 (frequent tapping) which is around 0.8 in the none-coupling condition (the area of R < 0.2, Figure 3).

But in this ANOVA result section, the dependent variable is the mean and SD of nITIs within a short 4-tap segment (over the 1st to 7th segement from the beginning), different from the whole trial sequence average we used for clustering. This means that we do not have moment-to-moment representation of frequent tapping in the current data. We agree with the reviewer that it would be exciting to elucidate the shift occurs gradually or suddenly when we have the next opportunity to explore this particular question.

As such, we have decided not to add further interpretation in the ANOVA results section to avoid introducing unnecessary confusion about two different metrics. Nonetheless, the interpretation about this piece of result is mentioned in the later discussion (p. 27).

p. 25, first paragraph: characterized by (not characterized with)
and “switched” is not a good word here. Consider rephrasing.

Thank you for this comment. We have rephrased the sentence using the word ‘transition’ in its place.

p. 25, second paragraph, first line: highly frequent tapping (not very frequent)

We changed the wording as suggested.

p. 26, lines 3-4: The sentence starting with “Most importantly, … “ is not clear. Please rephrase.

We changed the wording as suggested.

p. 26, second paragraph, line 3: What kind of underpinning?

Thank you for this comment. We have clarified what we mean by ‘underpinning’ here.

p. 26-27: It’s sometimes unclear what you mean by beat window – please define or repeat if defined earlier on in the manuscript.

We now rephrased it with ‘beat interval’ in p. 26-27.

p. 28, second paragraph (references). The following study shows that prior experiences (expert stylistic knowledge) influences beat bins and beat perception in general.

Danielsen, A., Nymoen, K., Langerød, M. T., Jacobsen, E., Johansson, M., & London, J. (2021). Sounds familiar (?): Expertise with specific musical genres modulates timing perception and micro-level synchronization to auditory stimuli. Attention, Perception, & Psychophysics, 1-17.

Thank you. This reference has been added.

p. 28, end of last complete paragraph: “ a motor process”

Thank you. This has been changed.

p. 29, second line: consider adding “…easily in all the conditions with some degree of coupling.”

Thank you for this comment. We have updated the sentence with the suggested phrase.

Reviewer #2: Thank you for carefully adrressing my comments. I just noticed a mistake in one reference (J. A. Grahn, p5)

Thank you for finding the reference typo. We corrected a few other mistakes from the reference manager software glitches.
