# Supplementary material for: Individual differences of limitation to extract beat from Kuramoto coupled oscillators: Transition from beat-based tapping to frequent tapping with weaker coupling
Source: PLoS One. 2023 Oct 9;18(10):e0292059. doi: 10.1371/journal.pone.0292059 (PMC10561847; doi:10.1371/journal.pone.0292059)
Supplement: Attachment — Submitted filename: comments-8-21-23.docx [file pone.0292059.s009.docx]

Dear reviewers,

We appreciate your detailed and insightful comments. We think that our manuscript has improved by taking into account your criticisms and suggestions. Below we respond to each of your comments and note where and how those responses are handled in the revised manuscript. Revisions to the manuscript and our responses to comments below are in blue font.

Reviewer #1: Thank you for carefully addressing my comments and suggestions. I have a few more minor comments/suggestions that I think would improve the paper. Given that these are addressed, I am happy to recommend the paper for publication and do not need to see another version.

Thank you for all your very insightful comments and we are pleased to hear you will recommend the paper for publication.

Abstract:
The new abstract better reflects the content of the article. However, make sure that the wording matches the text in the main manuscript (for example, use «highly frequent tapping» instead of «frequently tapped»). The use of the word «while» is also somewhat confusing. I also suggest asking a native speaker to do a copy edit of the abstract.

Thank you for the suggestion. We rephrased the wordings in those places.

P. 28: «Such criteria might be further informed by different prior experiences with or individual preferences for musical beats or metrical groupings (Jongsma, Desain, and Honing 2004; Snyder, Pasinski, and McAuley 2011; Danielsen et al. 2022).»

Nori Jacoby’s large-scale cross-cultural study of metrical priors would give further support to this point.

Reference:
Jacoby, N., Polak, R., Grahn, J., Cameron, D. J., Lee, K. M., Godoy, R., ... & McDermott, J. H. (2021). Universality and cross-cultural variation in mental representations of music revealed by global comparison of rhythm priors.

Thank you for this suggestion. We have added this reference in the manuscript in the appropriate place.

P. 31:
«Our results also confirmed our first hypothesis in that when the coupling is stronger, people show integrative processing of multi-onset information to achieve regular tapping which becomes more variable, consistent with the gradual spread of the perceptual beat-bin.»
Something missing after «becomes more variable» in this sentence? Perhaps change to «…becomes more variable with weaker coupling…»? I also suggest replacing «spread» with «widening».

Thank you for the suggestion. We rephrase the few sentences here to include the suggested wording for clarification (now p.41)

References: I noticed some inconsistencies in the style of the references. Make sure that all references are complete, consistently styled, and in line with the journal’s requirements.

Thank you for this suggestion. We have edited the sentence to provide more clarity. We have also changed the format of the manuscript’s citations to abide by the Vancouver citation format suggested by PLOS ONE and solved the reference managing software’s technical problems. Each citation was checked for its inclusion in the references section.
